# Alcohol Use Disorders and Increased Risk of Adverse Birth Complications and Outcomes: An 11-Year Nationwide Cohort Study

**DOI:** 10.3390/ijerph17228515

**Published:** 2020-11-17

**Authors:** Sarah Soyeon Oh, Yongho Jee, Eun-Cheol Park, Young Ju Kim

**Affiliations:** 1 Fetal Alcohol Syndrome Prevention Center, Ewha Mokdong Hospital, Ewha Womans University, Seoul 07985, Korea; sarahoh@ewha.ac.kr (S.S.O.); jyongho@ewha.ac.kr (Y.J.); 2 Department of Obstetrics & Gynecology, Ewha Womans University, Seoul 07985, Korea; 3 Advanced Biomedical Institute, Ewha Womans University Seoul Hospital, Seoul 07985, Korea; 4 Department of Preventive Medicine, Yonsei University, Seoul 03722, Korea; ecpark@yuhs.ac; 5 Institute of Health Services Research, Yonsei University, Seoul 03722, Korea

**Keywords:** preterm birth, prenatal alcohol exposure, birth complications

## Abstract

For women who suffer from Alcohol Use Disorders (AUDs), the use of alcohol before and/or during pregnancy may result in various birth complications, including miscarriage, stillbirth, or preterm delivery. Thus, this study aimed to explore whether Alcohol Use Disorders (AUDs) are associated with increased risk of adverse birth complications and outcomes. A total of 76,799 deliveries between 2003 and 2013 in the Korean National Health Insurance Service National Sample Cohort (NHIS-NSC) were analyzed. Women with an AUD diagnosis preceding delivery were identified as individuals with alcohol dependence. A multivariate Cox proportional hazards model was used to estimate the hazard ratio of adverse birth complications and outcomes associated with alcohol dependence. Diagnosis of an AUD was associated with increased risk of adverse birth complications (Hazard Ratio [HR]: 1.15, 95% CI: 1.01–1.31, *p* = 0.0302). This was especially the case for women whose AUD diagnosis was in the same year as their delivery (HR: 1.53, 95% CI: 1.24–1.88, *p* < 0.0001). AUDs were associated with increased risk of adverse birth outcomes, especially when prevalent in the same year as a woman’s delivery. Our study confirms that the monitoring of expecting women with a diagnosis of alcohol-related problems may be useful in preventing adverse birth complications.

## 1. Introduction

During the COVID-19 pandemic, alcohol consumption has been increasing as individuals experience increased feelings of social estrangement, self-isolation, and a sense of loneliness [1]. For individuals with Alcohol Use Disorders (AUDs), this proposes a serious health-related problem as chronic ethanol intake is associated with increased risk of bacterial and viral lung infections, including COVID-19 [1], as well as multisystemic damage to the liver, heart, lungs, and body [2].

AUDs are a maladaptive pattern of heavy and harmful alcohol use that leads to various behavioral, cognitive, and physiological phenomena, including a strong desire to drink, increased tolerance, and physical withdrawal [3]. Globally, the World Health Organization (WHO) estimates that the harmful use of alcohol is responsible for approximately 3 million deaths (5.3% of all deaths) each year [4]. In the United States alone, AUDs are believed to affect an estimated 15 million people, including 9.2 million men and 5.2 million women [5]. In South Korea, AUDs are believed to affect around 12.2% of adults between 18 and 74 years old, including one in five men [6].

For women who suffer from AUDs, the use of alcohol before and/or during pregnancy can result in various pregnancy and birth complications, including miscarriage, stillbirth, preterm delivery, and sudden infant death syndrome [7]. In one study of more than 3000 deliveries in the U.S., it was found that for every unit increase in alcohol exposure, the risk of preterm delivery increased by 34-fold [8]. In a meta-analysis study of 231,808 pregnant women, those exposed to alcohol during pregnancy had a greater risk of miscarriage (Odds Ratio [OR]: 1.19, 95% CI: 1.12–1.28), with each additional alcoholic drink consumed during pregnancy being associated with a 6% increase in miscarriage risk (OR: 1.06, 95% CI: 1.01–1.10) [8]. In an Ethiopian case-control study, mothers who drank alcohol during pregnancy were 7.56 times more likely to have a stillbirth compared to abstainers (OR: 7.56, 95% CI: 1.68–34.04) [9].

Because alcohol may cause an angiogenic imbalance that affects lipid levels, inflammation, and oxidative stress [10], drinking has also been associated with various clinical symptoms including hypertension, proteinuria, and intrauterine growth restriction (IUGR). For example, in a study of 76,940 pregnant women in Japan, alcohol consumption during pregnancy was associated with significantly higher odds of hypertensive disorders of pregnancy (OR: 3.98, 95% CI: 1.33–11.9), and placental accrete (OR: 3.10, 95% CI: 1.69–5.44) [11]. In another study of pregnant women exposed to alcohol and tobacco in Spain, the odds of IUGR increased by 22-fold, while the odds of small gestational age (SGA) increased by 33-fold [12].

While many published reports have focused on an association between alcohol consumption during pregnancy and such outcomes, few studies have focused on the association between a diagnosis of alcohol addiction preceding pregnancy and subsequent consequences. Despite the fact that the risk for alcohol use during pregnancy increases if the mother used and/or abused alcohol before her pregnancy [13], few studies have found a link between drinking before conception and adverse outcomes. Recently, one study found that parental binge drinking before conception may be associated with a baby’s congenital heart defects [14], possibly because alcohol exposure affects the DNA in developing sperm [15]. Another study claimed that alcohol intake before and during pregnancy is not associated with infant motor development, and is only slightly associated with mean infant weight, length, and head circumference at birth [16,17]. Because such studies are still new, there is no evidence concerning the effects of a previous alcohol diagnosis on adverse birth outcomes, especially for a nationwide population-based cohort.

Thus, this study aimed to examine the association between Alcohol Use Disorders (AUDs) and the risk of adverse birth complications and outcomes using nationwide cohort data.

## 2. Materials and Methods

The data used in this study were obtained from the National Health Insurance Service National Sample Cohort (NHIS-NSC) in Korea for the years 2002 to 2013. These data were constructed with the sole purpose of providing public health researchers and policy-makers with representative, useful information regarding Korean citizens’ utilization of health insurance and health examinations [18]. To construct the cohort, a representative sample cohort of 1,109,938 participants, representative of 2.2% of the Korean population, was selected using systematic stratified random sampling with proportional allocation of 1476 strata constructed based on age, sex, insurance eligibility status, and income [18]. Within each stratum, systematic sampling was conducted after sorting population data by the value of total annual medical expenses, and drawing stratum samples iteratively until an absolute percentage error of less than 5% was reached. During the follow-up period, a representative sample of newborns was added annually, and all deceased or emigrated participants were excluded [18,19]. The final population was followed for 11 years, during which a representative sample of newborns was added annually, and all deceased or emigrated participants were excluded [18].

For the identification of pregnancy, we employed the International Classification of Diseases, Tenth Revision, Clinical Modification (ICD-10-CM) diagnosis codes Z32.1, Z33, Z34.00, Z34.80, Z34.90, Z35, and O30, as seen in a previous study using the same dataset [19]. Incidence and risk of adverse birth complications (gestational hypertension, gestational diabetes, premature rupture of membranes (PROM), placenta previa, abruption placentae, postpartum hemorrhage, meconium stained amniotic fluid, fetal asphyxia, preterm birth, low birth weight, intrauterine growth restriction (IUGR) and outcomes (spontaneous/threatened/missed abortions, miscarriage) were identified using ICD-10 codes shown in the Supplementary Material Table S1. For accuracy purposes, only the first pregnancy of all women in our dataset was examined.

For the identification of AUDs, we employed the International Classification of Diseases, Tenth Revision, Clinical Modification (ICD-10-CM) diagnosis codes E24.4 (alcohol-induced pseudo-Cushing’s syndrome), F10 (mental and behavioural disorders due to use of alcohol), G31.2 (degeneration of nervous system due to alcohol), G62.1 (alcoholic polyneuropathy), G72.1 (alcoholic myopathy), I42.6 (alcoholic cardiomyopathy), K29.2 (alcoholic gastritis), K70 (alcoholic liver disease), K85.2 (alcohol-induced acute pancreatitis), K86.0 (alcohol-induced chronic pancreatitis), as seen in a previous study of alcohol use disorders [20].

We controlled for numerous patient-related covariates when analyzing the association between alcoholism and adverse birth outcomes. Such variables included age of delivery, income, insurance coverage, employment status, region, and year of diagnosis.

Age was divided into six 5 year groups (<20, 20–24, 25–29, 30–34, 35–39, ≥40) to reflect any differences in the association between alcoholism and adverse outcomes by maternal age at delivery. Insurance coverage was categorized into three groups: insurance (regional), insurance (corporate), and Medical Aid. Based on the criteria of South Korea’s National Health Insurance (NHI) system, individuals who are self-employed are covered by regional insurance, while individuals employed by a company are covered by employer-based corporate insurance. Medical aid beneficiaries consist of all those who have an income below the government-defined poverty threshold or a disability which enables them to receive free inpatient and outpatient care through the government.

Region was divided into three categories according to population density: metropolitan (Seoul, Gyeong-gido), city (Busan, Daegu, Incheon, Gwangju, Daejeon, Ulsan), and other (Gangwondo, Chungcheongbukdo, Chungcheongnamdo, Jeollabukdo, Jeollanamdo, Gyeongsangbukdo, Gyeongsangnamdo, Jeju Island).

We first examined the frequencies and percentages of each categorical variable at each patient’s baseline and performed χ^2^ tests to examine the distribution for adverse birth complications and outcomes according to each variable. Subsequently, we performed survival analyses using a Cox proportional hazards model to examine the factors significantly associated with adverse pregnancy outcomes. Cases with an event of birth complication or abortion were coded as 1, and all other cases were coded as 0. The follow-up period in the study was from 1 January 2003 to 31 December 2013 with the length of survival recorded in days. The HR and 95% confidence interval (CI) were computed from the Cox proportional hazard model, and statistical significance was determined by a two-tailed test with a *p*-value of 0.05 as the threshold. All statistical analyses were performed using SAS software, version 9.4 (SAS Institute, Cary, NC, USA).

All methods were performed in accordance with relevant guidelines and regulations. All experimental protocols were approved by the Institutional Review Board of Yonsei University’s Health System (Y-2019-0174). The need for informed consent was waived since the NHIS-NSC provides anonymous cohort data to researchers for scholarly use.

## 3. Results

This Table 1 presents the general characteristics of the study population. Of 76,799 women with confirmed pregnancies during our study period, 1211 (1.57%) were in the AUD group, and 75,588 (98.43%) were in the control group. Among those with AUDs, 19.7% had adverse outcomes, compared to 15.2% in the control group. Women with AUD diagnosis in the same year as their delivery had the highest prevalence of adverse birth outcomes (24.3%), while women with no AUD diagnosis had the lowest prevalence of adverse birth outcomes (15.2%).

Table 2 presents the results of the Cox proportional hazards model examining the association between AUD diagnoses, AUD diagnosis proximity to delivery, and adverse pregnancy outcomes. Women with a diagnosis of AUDs had increased risk (HR: 1.15, 95% CI: 1.01–1.31, *p* = 0.0302) of adverse birth outcomes, especially if her AUD diagnosis was made in the same year as her delivery (HR: 1.53, 95% CI: 1.24–1.88, *p* < 0.0001). Age at delivery was also significantly associated with increased adverse birth outcomes: compared to the 20–24 years’ age group, women who gave birth in the 25–29 age group (HR: 1.41, 95% CI: 1.30–1.53, *p* < 0.0001), 30–34 age group (HR: 1.61, 95% CI: 1.48–1.74, *p* < 0.0001), 35–39 age group (HR: 1.73, 95% CI: 1.58–1.89, *p* < 0.0001), and ≥40 age group (HR: 1.40, 95% CI: 1.21–1.61, *p* < 0.0001) had increased odds of adverse birth outcomes.

Working with corporate insurance coverage also had increased risk of adverse birth outcomes (HR: 1.06, 95% CI: 1.01–1.10, *p* = 0.0129) compared to women with regional insurance coverage, as did women who had already given birth 2 times (HR: 1.80, 95% CI: 1.57–2.06, *p* < 0.0001), three times (HR: 2.80, 95% CI: 2.46–3.20, *p* < 0.0001), and four or more times (HR: 2.70, 95% CI: 2.46–2.96, *p* < 0.0001). Women who gave twin births (HR: 1.57, 95% CI: 1.40–1.75, *p* < 0.0001) were at increased odds of adverse birth outcomes compared to women who gave single births also.

On the contrary, compared to women living in metropolitan regions, women living in the city (HR: 0.93, 95% CI: 0.89–0.97, *p* = 0.0010) and other regions (HR: 0.92, 0.88–0.96, *p* = 0.0001) had decreased risk of adverse birth outcomes.

Table 3 presents the results of the subgroup analysis of the association between types of adverse birth outcomes and AUD diagnosis. AUD diagnosis was most strongly associated with increasing risk of placenta previa (HR: 2.39, 95% CI: 0.97–5.91, *p* = 0.06) and Intrauterine Growth Restriction (HR: 1.77, 95% CI: 1.25–2.49, *p* = 0.00).

## 4. Discussion

Our findings show that AUD diagnosis is significantly associated with increased risk of adverse birth outcomes, especially when the diagnosis is made in close proximity to delivery (same year as delivery). To our knowledge, few studies to date have employed an ICD-10 diagnosis of AUDs as a marker for predicting adverse birth outcomes, despite the fact that self-reported measures of alcohol consumption during pregnancy are presumed to be highly unreliable.

Although instruments like the T-ACE/T-ACER-3, TWEAK, or Substance Use Risk Profile-Pregnancy (SURP-P) have been empirically validated for use among pregnant women [21], it is undeniable that self-reports are heavily influenced by recall bias. More importantly, because of the social stigmas surrounding substance use during pregnancy, it is highly likely that such self-reports are underestimates of actual alcohol consumption experience during pregnancy [22,23,24]. It should be noted that women who report drinking during pregnancy, but fail to provide exact information on the amount of alcohol they consumed during pregnancy, have the highest risk of stillbirths [7]. Such findings provide evidence that self-reports of alcohol consumption during pregnancy are heavily dependent on social desirability and stigmas.

Until more research on biomarkers like meconium ethyl glucuronide (EtG) are developed to detect alcohol consumption during pregnancy [24], employing ICD-10 diagnostic codes for AUDs may provide accurate information regarding serious, alcohol-related problems among pregnant women.

While evidence has been conflicting regarding the association between alcohol diagnosis prior to and during pregnancy and risk of adverse birth outcomes, one prospective cohort of 1303 pregnant women in the United Kingdom found that intakes of more than 2 units of alcohol/week before pregnancy, and in trimesters 1 and 2, were significantly associated with increased risk of babies with lower birth weight, lower birth centile, and preterm birth compared to non-drinkers [17]. Likewise, in a recent animal study in South Korea, alcohol consumption before or during pregnancy was associated with increased risk of abnormal fetal development in ethanol-fed mice, who not only had significantly decreased growth rates during the lactation period, but postnatal macrosomia [25,26]. Although these results were not replicated in our study, we also saw an inverse pattern between AUD diagnosis proximity to delivery and adverse birth outcomes. We were unable to control for various factors like exact alcohol intake (units/week) during pregnancy, and continued alcohol consumption during pregnancy due to the secondary nature of our data; however, controlling for these variables in future studies may result in similar results to the existing body of literature.

Like previous studies, our study also found an association between high number of births and the birth of twins with increased adverse birth outcomes. A high number of previous births has been associated with poor pregnancy outcomes in a number of studies [27,28], and in vitro fertilization twins are believed to have small but significantly increased risks of preterm birth and low birth weight [16].

Surprisingly, AUD diagnosis was not associated with increased risk of many individual birth complications: AUD diagnosis was only associated with increased risk of intrauterine growth retardation in our subgroup analysis. Many studies have explored the association between prenatal alcohol exposure and intrauterine growth retardation [29,30,31], including a study of pregnant rats in China, that found a significant association between alcohol consumption and reduced fetal developmental indices, placental weight, and oxidative and anti-oxidative functioning [32].

The limitations of our study derive from the limited accuracy and reliability of the ICD-10 classification of alcoholism within our study population. Although it must be assumed that the classifications were made by healthcare professionals fully aware and abiding of specific criteria for maximum diagnostic reliability, chronic alcoholism is a difficult condition to diagnose. Furthermore, diagnosing alcoholism among individuals with disability can be extremely challenging for clinicians; heavy drinking associated with alcoholism can contribute to, or result from, several different psychiatric syndromes ranging from depression to sociopathy and dementia, that make diagnosis confusing and inaccurate [33].

Second, our study centered on the time until adverse birth outcome following year of diagnosis (2003), which was around ten years. Therefore, during this time period, various confounding variables will have influenced the patient’s risk of adverse birth outcomes, relative to medical advancements, seasonal biological changes, and social, economic, and political factors. As of yet, there are not enough previous studies with regard to a nationally representative population of Koreans when it comes to measuring the impact of alcoholism and adverse birth outcomes. It is difficult to see whether the figures we calculated are similar to that of the statistics found in previous studies for Koreans.

Also, while our study specifically focused on adverse birth outcomes that occur during birth and mostly affect the mother, further studies should also attempt to examine the long-term and more severe consequences of prenatal alcohol exposure on child development, i.e., fetal alcohol spectrum disorders (FASDs). Prenatal alcohol exposure has been associated with a range of mental and/or physical disabilities among individuals whose mothers consumed alcohol during pregnancy [34]. Previous studies have stated that consuming alcohol during pregnancy is one of the “most verified” prenatal risk factors for impaired child development [24]. More studies are required to understand how AUD diagnosis may affect the prevalence of such diseases as it is believed that FASDs occur among one in 13 prenatally exposed infants [35].

Lastly, while we assumed that alcohol consumption during pregnancy would be prevalent if an AUD diagnosis was made in the same year as delivery, it is impossible to know whether or not this was so. This was a limitation of our secondary data, but it is highly recommended that future studies employ instruments like the T-ACE/T-ACER-3, TWEAK, or Substance Use Risk Profile-Pregnancy (SURP-P) in simultaneity with AUD diagnoses, for a more accurate prediction of alcohol consumption during pregnancy [21].

Despite these limitations, our study has a number of strengths. It is one of the first reports to address the association between AUD diagnosis and adverse birth complications, while controlling for the proximity between diagnosis time and birth. To our knowledge, no study in South Korea has attempted to evaluate the association between alcohol consumption during pregnancy and adverse birth outcomes, mostly because data on alcohol consumption during pregnancy are very rare, if not non-existent, in our country as of now. Furthermore, our data consisted of a nationally representative sample of women who were examined by medical professionals for the NHIS database. Most importantly, instead of using retrospective self-reports of alcohol consumption during pregnancy, we measured the possibility for alcohol-related problems during pregnancy through an ICD-10 diagnosis of AUDs, which allowed for an accurate identification of expecting women with past/present alcohol-related problems.

## 5. Conclusions

Overall, such findings suggest that AUDs are significantly associated with increased risk of adverse birth outcomes, especially when prevalent in the same year as a woman’s delivery. It is highly recommended that healthcare professionals routinely ask all pregnant women about their alcohol consumption, and pay special attention to women with a previous diagnosis of AUDs.

Validated behavioral interventions for AUDs in pregnancy include motivational enhancement therapy and cognitive behavior therapies such as brief psychodynamic psychotherapy, interpersonal therapy, educational interventions, and supportive counseling [21]. Pharmacological treatments with medications like benzodiazepines and clomethiazole are not recommended for pregnant women because of the risk of possible teratogenic effects [36]. Likewise, although naltrexone has shown potential for use among pregnant, alcohol-dependent women, pharmacological treatments of any kind should only be considered as a treatment option after a clinician has carefully evaluated the implications of medication use on both the mother and fetus.

As AUDs are associated with neurobiological adaptations that reduce an individual’s cognitive control, clinicians must be non-judgmental, non-directive, and private [37,38]. Empathy and mutual respect are also important as previous research has found a positive association between these factors and recovery from substance abuse among pregnant women [39]. Further research on preventive measures, as well as pharmacological treatment of alcohol abuse and/or dependence among pregnant women, is necessary to prevent adverse birth complications and outcomes among this population. Lastly, healthcare professionals should always be supported by comprehensive, up-to-date information on prenatal alcohol use and incorporate such information to prevent alcohol use among women *before* they become pregnant [40].

## Figures and Tables

**Table 1:** General Characteristics of Deliveries and Adverse Pregnancy Outcome by AUD Diagnosis & Diagnosis Proximity to Delivery.

Variables	Total Deliveries	None		Adverse Outcomes		*p*-Value
		*n*	%	*n*	%	
**Alcohol Use Disorder (AUD) Diagnosis**						
None	75,588	64,130	84.8	11,458	15.2	<0.0001
Yes	1211	972	80.3	239	19.7	
**AUD Diagnosis Proximity to Delivery**						
Same year as delivery	370	280	75.7	90	24.3	<0.0001
1 year before delivery	208	170	81.7	38	18.3	
2 years before delivery	146	120	82.2	26	17.8	
≥3 years before delivery	487	402	82.5	85	17.5	
None	75,588	64,130	84.8	11,458	15.2	
**Age at Delivery**						
<20	842	794	94.3	48	5.7	<0.0001
20–24	6924	6223	89.9	701	10.1	
25–29	29,745	25,313	85.1	4,432	14.9	
30–34	28,604	23,816	83.3	4,788	16.7	
35–39	8483	7020	82.8	1463	17.2	
≥40	2201	1936	88.0	265	12.0	
**Income**						
Low	9439	8088	85.7	1351	14.3	0.0012
Medium	45,588	38,696	84.9	6892	15.1	
High	21,772	18,318	84.1	3454	15.9	
**Employment Status**						
Unemployed	48,447	41,333	85.3	7114	14.7	<0.0001
Employed	28,352	23,769	83.8	4583	16.2	
**Medical Insurance**						
Insurance Coverage (Regional)	23,828	20,553	86.3	3275	13.7	<0.0001
Insurance Coverage (Corporate)	52,674	44,280	84.1	8394	15.9	
Medical Aid	297	269	90.6	28	9.4	
**Disability**						
None	76,373	64,736	84.8	11,637	15.2	<0.0001
Moderate	317	266	83.9	51	16.1	
Severe	109	100	91.7	9	8.3	
**Number of Births**						
1	7304	6814	93.3	490	6.7	<0.0001
2	3413	3038	89.0	375	11.0	
3	2549	2141	84.0	408	16.0	
≥4	63,533	53,109	83.6	10,424	16.4	
**Twin Birth Status**						
Single Birth	75,468	64,096	84.9	11,372	15.1	<0.0001
Twin Birth	1331	1006	75.6	325	24.4	
**Region**						
Metropolitan	35,844	30,076	83.9	5768	16.1	<0.0001
City	19,265	16,433	85.3	2832	14.7	
Other	21,690	18,593	85.7	3097	14.3	
**Year of Diagnosis**						
2003	14,311	12,890	90.1	1421	9.9	<0.0001
2004	9127	7937	87.0	1190	13.0	
2005	7975	6927	86.9	1048	13.1	
2006	7069	6069	85.9	1000	14.1	
2007	7018	5953	84.8	1065	15.2	
2008	5719	4702	82.2	1017	17.8	
2009	5100	4209	82.5	891	17.5	
2010	5486	4398	80.2	1088	19.8	
2011	5313	4246	79.9	1067	20.1	
2012	4972	3865	77.7	1107	22.3	
2013	4709	3906	82.9	803	17.1	
	76,799	65,102	84.8	11,697	15.2	

**Table 2:** Survival Analysis of Association between AUD Diagnosis and Adverse Birth Outcomes.

	Adverse Birth Outcomes			
	Hazard Ratio (HR)	95% CI		*p*-Value
		Lower	Upper	
**Alcohol Use Disorder (AUD) Diagnosis**				
None	1.00	-	-	
Yes	1.15	1.01	1.31	0.0302
**AUD Diagnosis Proximity to Delivery**				
Same year as delivery	1.53	1.24	1.88	<0.0001
1 year before delivery	1.17	0.85	1.60	0.3461
2 years before delivery	0.99	0.67	1.46	0.9644
≥3 years before delivery	0.95	0.76	1.17	0.6142
None	1.00	-	-	
**Age at Delivery**				
<20	0.58	0.43	0.77	0.0002
20–24	1.00	-	-	
25–29	1.41	1.30	1.53	<0.0001
30–34	1.61	1.48	1.74	<0.0001
35–39	1.73	1.58	1.89	<0.0001
≥40	1.40	1.21	1.61	<0.0001
**Income**				
Low				
Medium	0.98	0.91	1.04	0.4551
High	0.97	0.93	1.02	0.2268
**Employment Status**				
Unemployed	1.00	-	-	
Employed	1.00	0.96	1.04	0.9079
**Medical Insurance**				
Insurance Coverage (Regional)	1.00	-	-	
Insurance Coverage (Corporate)	1.06	1.01	1.10	0.0129
Medical Aid	0.79	0.54	1.15	0.2173
**Disability**				
None	1.00	-	-	
Moderate	1.06	0.81	1.40	0.6789
Severe	0.60	0.31	1.16	0.1274
**Number of Births**				
1	1.00	-	-	
2	1.80	1.57	2.06	<0.0001
3	2.80	2.46	3.20	<0.0001
≥4	2.70	2.46	2.96	<0.0001
**Twin Birth Status**				
Single Birth	1.00	-	-	
Twin Birth	1.57	1.40	1.75	<0.0001
**Region**				
Metropolitan	1.00	-	-	
City	0.93	0.89	0.97	0.0010
Other	0.92	0.88	0.96	0.0001
**Year of Diagnosis**				
2003	1.00	-	-	
2004	1.33	1.23	1.43	<0.0001
2005	1.35	1.24	1.46	<0.0001
2006	1.44	1.33	1.56	<0.0001
2007	1.57	1.45	1.71	<0.0001
2008	1.91	1.76	2.07	<0.0001
2009	1.92	1.76	2.09	<0.0001
2010	2.31	2.14	2.51	<0.0001
2011	2.37	2.19	2.57	<0.0001
2012	2.75	2.53	2.97	<0.0001
2013	3.59	3.29	3.93	<0.0001

**Table 3:** Subgroup Analysis of Association between Types of Adverse Birth Outcomes and AUD Diagnosis.

	None	Alcohol Use Disorder (AUD) Diagnosis *			
		Hazard Ratio (HR)	95% CI		*p*-Value
			Lower	Upper	
**Birth Complications**					
Gestational hypertension	1.00	0.62	0.09	4.50	0.64
Gestational diabetes	1.00	0.85	0.61	1.19	0.34
PROM *	1.00	0.95	0.55	1.64	0.85
Placenta previa	1.00	2.39	0.97	5.91	0.06
Abruptio placentae	1.00	-			
Postpartum hemorrhage	1.00	1.48	0.76	2.86	0.25
Meconium stained amniotic fluid	1.00	-			
Fetal asphyxia	1.00	-			
Preterm birth	1.00	1.27	0.82	1.97	0.28
Low birth weight	1.00	-			
IUGR **	1.00	1.77	1.25	2.49	0.00
**Birth Outcomes**					
Abortion	1.00	1.27	0.97	1.66	0.08
Miscarriage	1.00	1.14	0.95	1.37	0.15

* Premature rupture of membranes. ** Intrauterine Growth Restriction.

## Supplementary Materials

The following are available online at https://www.mdpi.com/1660-4601/17/22/8515/s1, Table S1: ICD-10 codes for pregnancy and adverse birth complications.

## References

[1] Testino G. (2020). Are Patients With Alcohol Use Disorders at Increased Risk for Covid-19 Infection?. Alcohol Alcohol..

[2] Ojo A.S., Akin-Onitolo A., Okediji P., Balogun S. (2020). COVID-19 and Alcoholism: A Dangerous Synergy?. J. Contemp. Stud. Epidemiol. Public Health.

[3] Rehm J. (2016). How should prevalence of alcohol use disorders be assessed globally?. Int. J. Methods Psychiatr. Res..

[4] WHO (2019). Global Status Report on Alcohol and Health 2018.

[5] Buechler H.M. (2020). Searching for new medications for the treatment of alcohol use disorder. Theses and Dissertations.

[6] Jang J.Y., Kim D.J. (2018). Epidemiology of alcoholic liver disease in Korea. Clin. Mol. Hepatol..

[7] Bailey B.A., Sokol R.J. (2011). Prenatal alcohol exposure and miscarriage, stillbirth, preterm delivery, and sudden infant death syndrome. Alcohol Res. Health.

[8] Sundermann A.C., Zhao S., Young C.L., Lam L., Jones S.H., Velez Edwards D.R., Hartmann K.E. (2019). Alcohol Use in Pregnancy and Miscarriage: A Systematic Review and Meta-Analysis. Alcohol. Clin. Exp. Res..

[9] Tasew H., Zemicheal M., Teklay G., Mariye T. (2019). Risk factors of stillbirth among mothers delivered in public hospitals of Central Zone, Tigray, Ethiopia. Afr. Health Sci..

[10] Agarwal A., Aponte-Mellado A., Premkumar B.J., Shaman A., Gupta S. (2012). The effects of oxidative stress on female reproduction: A review. Reprod. Biol. Endocrinol..

[11] Cahill P.A., Redmond E.M. (2012). Alcohol and cardiovascular disease—Modulation of vascular cell function. Nutrients.

[12] Sabra S., Malmqvist E., Almeida L., Gratacos E., Gomez Roig M.D. (2018). Differential correlations between maternal hair levels of tobacco and alcohol with fetal growth restriction clinical subtypes. Alcohol.

[13] Lamy S., Houivet E., Marret S., Hennart B., Delavenne H., Benichou J., Allorge D., Thibaut F. (2019). Perinatal Network of Upper Normandy. Risk factors associated to tobacco and alcohol use in a large French cohort of pregnant women. Arch. Women’s Ment. Health.

[14] Zhang S., Wang L., Yang T., Chen L., Zhao L., Wang T., Chen L., Ye Z., Zheng Z., Qin J. (2019). Parental alcohol consumption and the risk of congenital heart diseases in offspring: An updated systematic review and meta-analysis. Eur. J. Prev. Cardiol..

[15] Savitz D.A., Schwingl P.J., Keels M.A. (1991). Influence of paternal age, smoking, and alcohol consumption on congenital anomalies. Teratology.

[16] McDonald S.D., Han Z., Mulla S., Ohlsson A., Beyene J., Murphy K.E. (2010). Preterm birth and low birth weight among in vitro fertilization twins: A systematic review and meta-analyses. Eur. J. Obstet. Gynecol. Reprod. Biol..

[17] McDonald B.W., Watson P.E. (2020). Maternal alcohol intakes before and during pregnancy: Impact on the mother and infant outcome to 18 months. Nord. Stud. Alcohol Drugs.

[18] Lee J., Lee J.S., Park S.-H., Shin S.A., Kim K. (2017). Cohort profile: The national health insurance service–national sample cohort (NHIS-NSC), South Korea. Int. J. Epidemiol..

[19] Moon H.-Y., Kim M.-R., Hwang D.-S., Jang J.-B., Lee J., Shin J.-S., Ha I., Lee Y. (2020). Safety of acupuncture during pregnancy: A retrospective cohort study in Korea. Bjog Int. J. Obstet. Gynaecol..

[20] Lid T.G., Eide G.E., Dalen I., Meland E. (2016). Can routine information from electronic patient records predict a future diagnosis of alcohol use disorder?. Scand. J. Prim. Health Care.

[21] DeVido J., Bogunovic O., Weiss R.D. (2015). Alcohol use disorders in pregnancy. Harv. Rev. Psychiatry.

[22] Burns L., Mattick R.P., Cooke M. (2006). Use of record linkage to examine alcohol use in pregnancy. Alcohol. Clin. Exp. Res..

[23] Czeizel A., Petik D., Puho E. (2004). Smoking and alcohol drinking during pregnancy. The reliability of retrospective maternal self-reported information. Cent. Eur. J. Public Health.

[24] Eichler A., Grunitz J., Grimm J., Walz L., Raabe E., Goecke T.W., Beckmann M.W., Kratz O., Heinrich H., Moll G.H. (2016). Did you drink alcohol during pregnancy? Inaccuracy and discontinuity of women’s self-reports: On the way to establish meconium ethyl glucuronide (EtG) as a biomarker for alcohol consumption during pregnancy. Alcohol.

[25] Ouidir M., Buck Louis G.M., Kanner J., Grantz K.L., Zhang C., Sundaram R., Rahman M.L., Lee S., Kannan K., Tekola-Ayele F. (2020). Association of Maternal Exposure to Persistent Organic Pollutants in Early Pregnancy With Fetal Growth. JAMA Pediatrics.

[26] Lee Y.J., Kim J.Y., Lee D.Y., Park K.J., Kim G.H., Kim J.E., Roh G.S., Lim J.Y., Koo S., Lim N.K. (2020). Alcohol consumption before pregnancy causes detrimental fetal development and maternal metabolic disorders. Sci. Rep..

[27] Grant T., Graham J.C., Ernst C.C., Peavy K.M., Brown N.N. (2014). Improving pregnancy outcomes among high-risk mothers who abuse alcohol and drugs: Factors associated with subsequent exposed births. Child. Youth Serv. Rev..

[28] Stover J., Ross J. (2013). Changes in the distribution of high-risk births associated with changes in contraceptive prevalence. BMC Public Health.

[29] Ernhart C.B., Wolf A.W., Linn P.L., Sokol R.J., Kennard M.J., Filipovich H.F. (1985). Alcohol-related birth defects: Syndromal anomalies, intrauterine growth retardation, and neonatal behavioral assessment. Alcohol. Clin. Exp. Res..

[30] Lundsberg L.S., Bracken M.B., Saftlas A.F. (1997). Low-to-moderate gestational alcohol use and intrauterine growth retardation, low birthweight, and preterm delivery. Ann. Epidemiol..

[31] Yang Q., Witkiewicz B.B., Olney R.S., Liu Y., Davis M., Khoury M.J., Correa A., Erickson J.D. (2001). A case-control study of maternal alcohol consumption and intrauterine growth retardation. Ann. Epidemiol..

[32] Li Y., Yan Y.-E., Wang H. (2011). Enhancement of placental antioxidative function and P-gp expression by sodium ferulate mediated its protective effect on rat IUGR induced by prenatal tobacco/alcohol exposure. Environ. Toxicol. Pharmacol..

[33] Black J.J., Heffner J.L., Anthenelli R.M., Beavers J.N., Albertz A., Blom T., Adler C., DelBello M.P. (2012). Diagnosing alcohol and cannabis use disorders in adolescents with bipolar disorder: A preliminary investigation. J. Dual Diagn..

[34] Oh S.S., Kim Y.J., Jang S.-i., Park S., Nam C.M., Park E.-C. (2020). Hospitalizations and mortality among patients with fetal alcohol spectrum disorders: A prospective study. Sci. Rep..

[35] Burd L. (2016). FASD and ADHD: Are they related and How?. BMC Psychiatry.

[36] Heberlein A., Leggio L., Stichtenoth D., Hillemacher T. (2012). The treatment of alcohol and opioid dependence in pregnant women. Curr. Opin. Psychiatry.

[37] Bakhireva L.N., Shrestha S., Garrison L., Leeman L., Rayburn W.F., Stephen J.M. (2018). Prevalence of alcohol use in pregnant women with substance use disorder. Drug Alcohol Depend..

[38] Martin R., Bruxner G., Ng G., Brewster C., Kothari A. (2020). Drowning our sorrows: Clinical and ethical considerations of termination in alcohol-affected pregnancy. BMC Pregnancy Childbirth.

[39] Motz M., Pepler D.J., Psych C., Moore T.E., Freeman P. (2005). Breaking the cycle: Measures of progress. J. FAS Int..

[40] France K., Henley N., Payne J., D’Antoine H., Bartu A., O’Leary C., Elliott E., Bower C. (2010). Health professionals addressing alcohol use with pregnant women in Western Australia: Barriers and strategies for communication. Subst. Use Misuse.

