# Supplementary material for: Alcohol Use Disorders and Increased Risk of Adverse Birth Complications and Outcomes: An 11-Year Nationwide Cohort Study
Source: Int J Environ Res Public Health. 2020 Nov 17;17(22):8515. doi: 10.3390/ijerph17228515 (PMC7698577; doi:10.3390/ijerph17228515)
Supplement: Supplementary file 1 [file ijerph-17-08515-s001.pdf]

Table S1. ICD-10 codes for pregnancy and adverse birth complications.

Variables	ICD-10* Code
**Pregnancy**	
Pregnancy confirmed	Z32.1
Pregnant state, incidental	Z33
Supervision of normal first pregnancy, less than 24 weeks pregnant	Z34.00
Supervision of other normal pregnancy, less than 24 weeks pregnant	Z34.80
Supervision of normal pregnancy, unspecified, less than 24 weeks pregnant	Z34.90
Supervision of high-risk pregnancy	Z35
Multiple gestation	O30
**Adverse Birth Complications**	
Gestational hypertension	O13
Gestational diabetes	O24
PROM (premature rupture of membranes)	O42
Placenta previa	O44
Abruptio placentae	O45
Postpartum hemorrhage	O72
Meconium stained amniotic fluid	P96
Fetal asphyxia	P21
Preterm birth	O60 (O600, O601, O602, O603)
Low birth weight	P07
IUGR (intrauterine growth retardation)	O365
**Adverse Birth Complications**	
Complete or unspecific spontaneous abortion without complication	O03.4
Incomplete spontaneous abortion without complication	O03.9
Missed abortion	O20.1
Threatened abortion	O02.0

*International Classification of Diseases, Tenth Revision
